# Skeletal Dysplasias That Cause Thoracic Insufficiency in Neonates: Illustrative Case Reports

**DOI:** 10.1097/MD.0000000000003298

**Published:** 2016-04-08

**Authors:** Mehmet Sah İpek, Cihan Akgul Ozmen

**Affiliations:** From the Department of Neonatology (MSİ), Maternity and Children's Hospital; and Department of Radiology (CA), Dicle University School of Medicine, Diyarbakir, Turkey.

## Abstract

Skeletal dysplasias are a heterogeneous group of conditions associated with various abnormalities of the skeleton. Some of them are perinatally lethal and can be diagnosed at birth. Lethality is usually due to thoracic underdevelopment and lung hypoplasia. A correct diagnosis and typing of the skeletal disorder is essential for the prognosis as is genetic counseling of the family. A retrospective review of 12 cases of clinico-radiologic diagnosis of skeletal dysplasia, leading to thoracic insufficiency, was conducted.

We aimed to make differential diagnosis with special emphasis on radiological findings, and to emphasize the importance of parental counseling.

## INTRODUCTION

Skeletal dysplasias are a large heterogeneous group of disorders consisting of abnormalities of bone or cartilage growth or their structure. Recently, 436 different entities have been described, despite the fact that the total number has gone down compared with the previous revision (by grouping of phenotypically indistinguishable entities).^1^ Although certain dysplasias individually are quite rare, their overall prevalence as a group has been reported to be 2.3 to 7.6 per 10,000 births in various epidemiologic studies.^2–4^ Some of them are perinatally lethal and can be diagnosed at birth, whereas some others are nonlethal and compatible with short or long-term survival.^4,5^ Approximately one-fifth of affected fetuses are stillborn, whereas one-third die during the first week of life.^4,5^ Lethality in these entities is usually due to thoracic underdevelopment and lung hypoplasia.^5^ Extensive vertebral and rib anomalies may result in thoracic cage deformity with adverse effects on thoracic growth and function.^6^ The inability of the thorax to support normal respiration or lung growth leads to thoracic insufficiency.^6^

Because of the considerable heterogeneity, the diagnosis of a skeletal dysplasia can be difficult.^5^ Despite advances in molecular genetics, as yet, there is no simple test available to achieve a diagnosis. Family history, and clinical and radiographic assessment all contribute to achieving a diagnosis. Radiographic evaluation is a common primary investigation in many diseases and is part of the skeletal survey for investigation of skeletal dysplasia. The initial identification and the decision to refer a patient for further molecular analysis and expensive genetic tests still frequently relies on clinical and radiological criteria.^5–9^

In this series, a review of 12 cases of clinico-radiologic diagnosis of skeletal dysplasia, which leads to thoracic insufficiency, is conducted. Thus, we intend to emphasize the examination of the skeletal radiographs based on the analysis of cardinal criteria, from which the most useful information is derived and which make further differentiation possible. Secondly, we would like to emphasize the importance of parental counseling, in accordance with that learned from case studies.

## METHODS

The database of our hospital, which is a maternity and children's hospital, was screened for neonates diagnosed with skeletal dysplasia from January 2011 to December 2014. Twenty-six patients were found. Of them, 15 showed respiratory distress/insufficiency during their hospital course. All hospital records including radiographs of them were reviewed. Two experienced radiologists independently evaluated all available radiographs of the patients. Clinico-radiologic diagnosis of skeletal dysplasia was precisely established in 12 patients. Unfortunately, genetic tests could not be performed due to the retrospective nature of the study. Ethical approval was not required because this is a retrospective case report based on medical records according to the guidelines of the Ministry of Health. Patient consent was not obtained because the study does not include any intervention or private patient information.

## RESULTS

**FIGURE 1:**
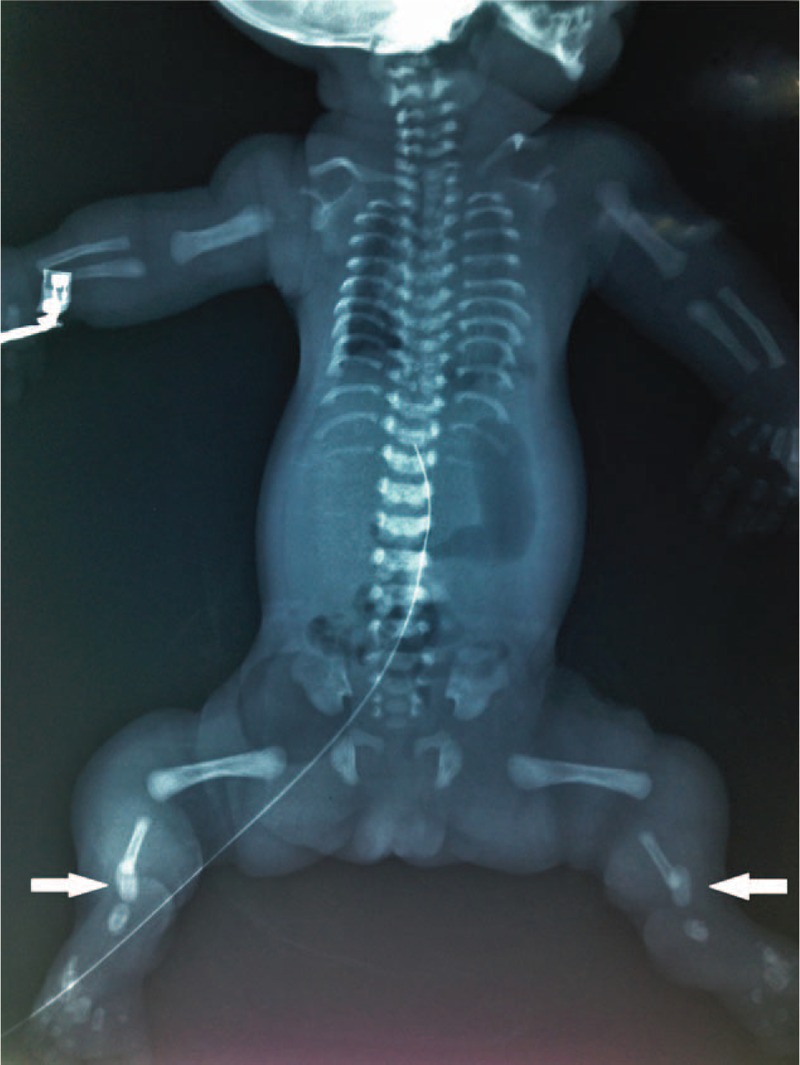
Radiograph of case 1 shows narrow thorax due to short and horizontally oriented ribs. The upper and lower limb long tubular bones are short. The pelvic appearance is normal. The metaphyseal ends of the long bones are rounded and there are extremely short ovoid tibiae (arrow).

**FIGURE 2:**
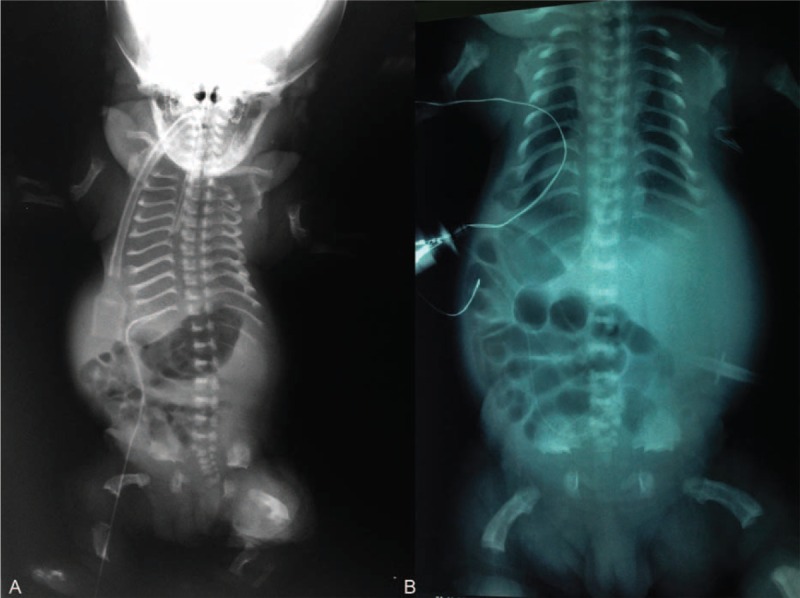
Radiographs of case 2 (A) and case 3 (B) show small thorax, and short and horizontal ribs with cupped anterior ends. The calvarium is relatively large (A). The humeri are curved and short. Platyspondyly with an H appearance of the vertebrae is noted. The ilia are small and squared, with a horizontal, trident-shaped acetabular roof. The greater sciatic notches are very small. The ischia and pubis are small, with widening of the symphysis and the sacroiliac joints. The femora show a “telephone receiver” shape. The femora are widened proximally with an oval area of decreased opacity similar to that seen in newborns with achondroplasia.

**FIGURE 3:**
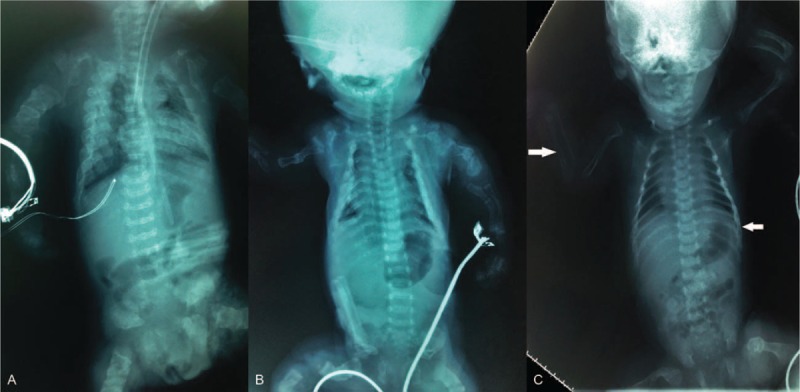
Radiographs of case 4 (A) and case 5 (B) show generalized undermineralization, short-limb dwarfism, rhizomelic limb shortening, small chest, and platyspondyly. Periosteal new bone in multiple healing fractures gives the ribs a beaded appearance. The ribs show continuous beading (A). All long bones are short, squared, and broad with crumpled shafts (due to multiple fractures with callus formation). Note “telescoped” or “accordion-like” appearance of femurs and humeri. The skull is poorly ossified (B). Note that bone changes in case 4 (A) are more severe than that in case 5 (B). In case 5, the ribs show multiple fractures in a discontinuous pattern with normal rib parts in between callus formation (discontinuous beading), and platyspondyly is less severe. In radiograph of case 6 (C), skull is large relative to facial bones, and calvarium is underossified. Biparietal diameter is increased. Bones of extremities are relatively long and thin, with bowing deformities and wide metaphyses. Ribs are thin, without overt fractures. Several fractures with callus formation are seen in rib, clavicles, and left femur. Two fractures without callus formation are seen in right radius and left 9th rib. Note radioulnar synostosis between the proximal radius and the ulna.

**FIGURE 4:**
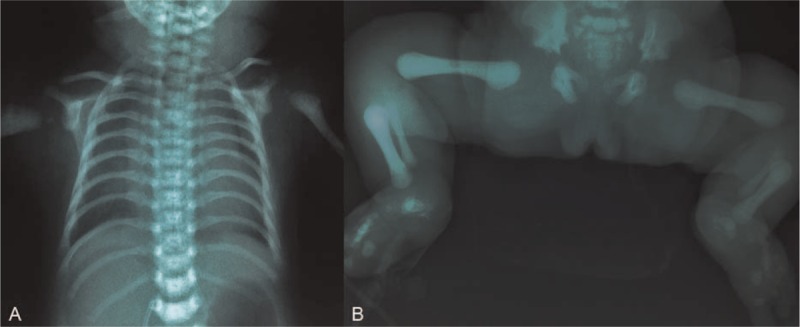
Frontal (A) chest radiograph of case 7 shows narrow thorax with slightly short ribs. The cardiomegaly is due to cardiac defect including atrial septal defect, ventricular septal defect, and patent ductus arteriosus. The vertebral bodies are unremarkable. Radiograph of lower limbs and pelvis (B) shows vertically shortened ilia, and their inferior margins, which are horizontal with a downward-pointing hook at the medial and lateral aspects of the acetabulae (trident or triradiate acetabulum). Femora and tibiae are short and broad, and their lower ends and proximal ends of tibiae are clubbed. Note that there is progressive distal shortening of limbs leading to mesomelia and acromelia. The appearance of the chest and pelvis closely resembles that of asphyxiating thoracic dystrophy.

**FIGURE 5:**
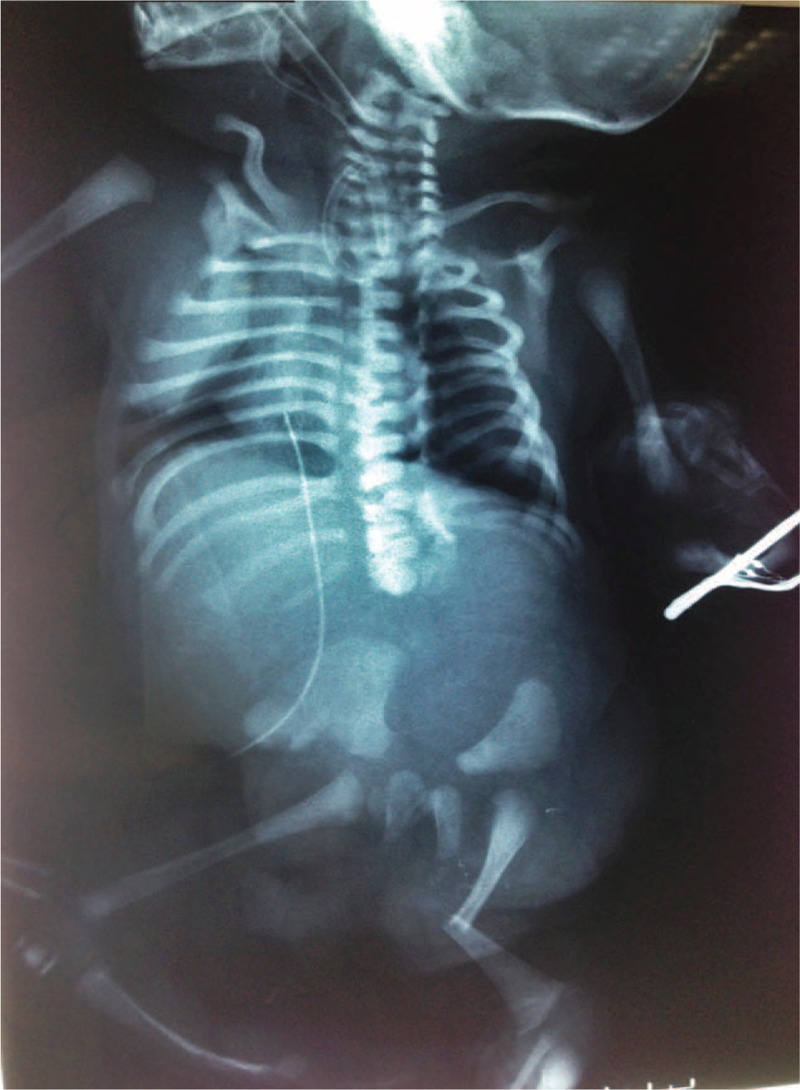
Radiograph of case 9 shows absence of lumbosacral vertebrae, thoracic vertebral defects, malformed ribs, dysplastic and hypoplastic pelvic bones, and “frog-like” position of the right lower extremity. Spine terminates at the T12 level. The left radius is absent (the absence of left thumb is not well visualized). The orogastric tube does not pass through esophagus, and is twisted. There is no passage of gas into the stomach and intestines. Note shaft fracture of the left femur, and dislocated left knee.

**FIGURE 6:**
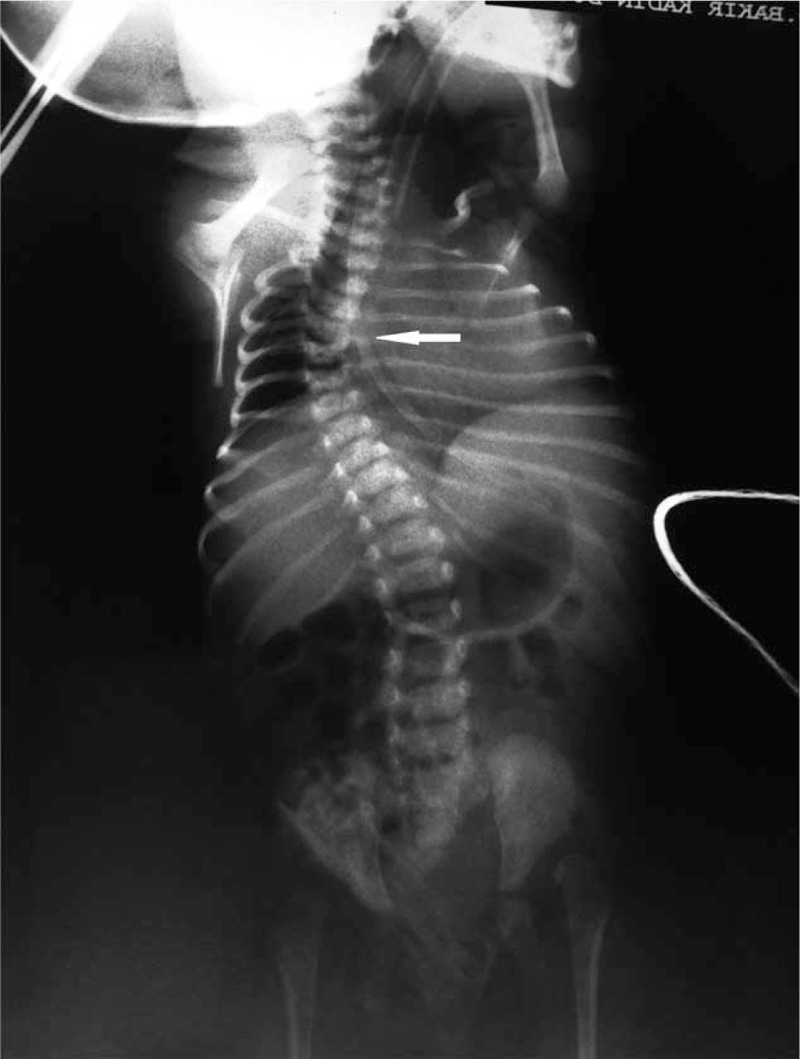
Radiograph of case 10 demonstrates scoliosis in the thoracic spine and reduced lung volume. Note that there is unilateral unsegmented bar (arrow), and vertebrae at the curve region are smaller. Bilateral dislocation of hip is also seen.

**FIGURE 7:**
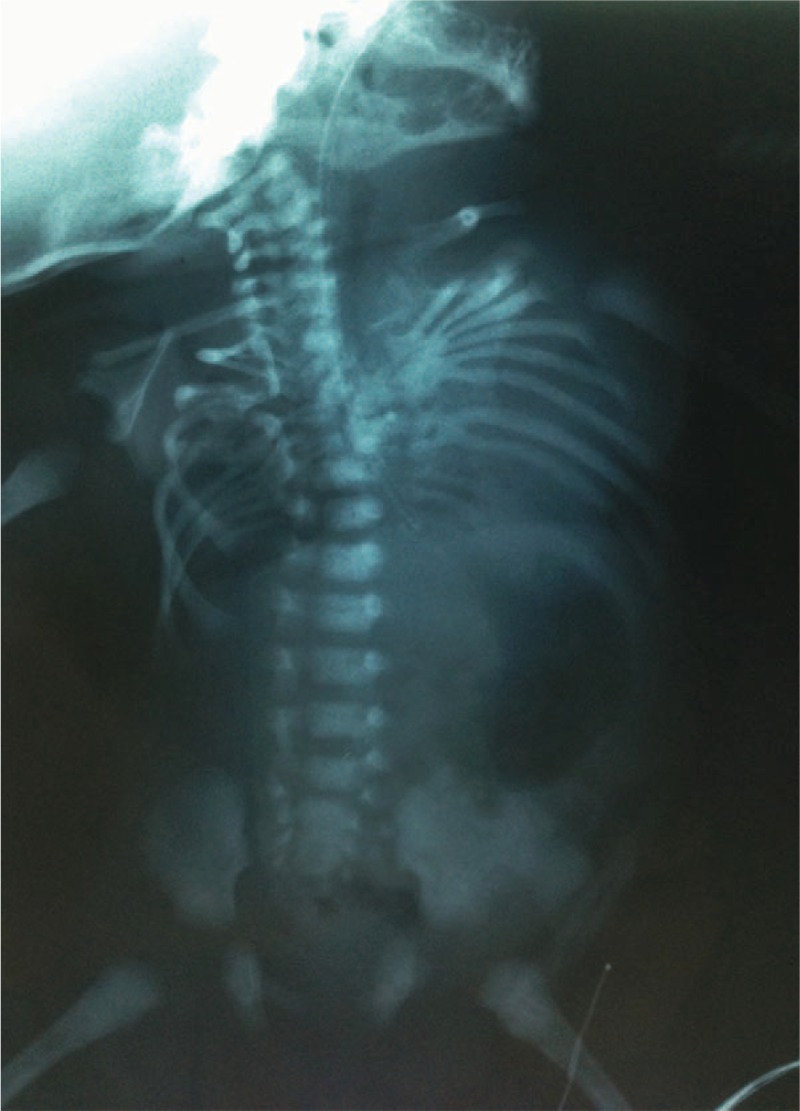
Radiograph of case 11 shows multiple abnormalities of the thoracic vertebrae with hemivertebrae, fused vertebrae, and fusion and bifurcation of the ribs. Segmentation anomalies appear throughout the majority of thoracic spine. Thorax is asymmetrical, and has mild scoliosis. The right lung is somewhat smaller than the left. Note that despite multiple vertebral segmentation defects, a “crab-like” thoracic spine is not evident. The radiograph shows asymmetrical abnormalities of the vertebral bodies and ribs, but not the symmetrical posterior fusion of the ribs so characteristic of spondylothoracic dysplasia.

**FIGURE 8:**
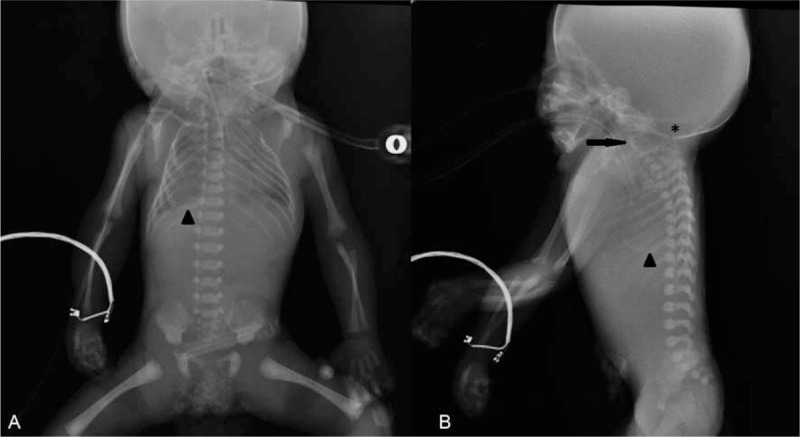
Frontal (A) radiograph of case 12 shows asymmetrical thoracic cage, and thin and deformed ribs. Sprengel deformity (congenital elevation of the scapula) is bilaterally present. Note that there is no passage of gas into the stomach and intestines. The orogastric tube does not pass through esophagus into the stomach, and is twisted in the thoracic cage (arrowhead). Lateral spine radiograph (B) shows fusion of adjacent cervical vertebral bodies (arrow), moderate dorsiflexion of cervical spine, and wide foramen magnum (asterisk). The calvarium is relatively large.

Clinico-radiologic diagnosis of skeletal dysplasia in 12 patients were as follows: short-rib polydactyly syndrome (SRPS) type 2 (Majewski syndrome; Figure 1), thanatophoric dysplasia (TD) type I (Figures 2A and B), osteogenesis imperfecta (OI) type IIA (Figure 3A), OI type IIB (Figure 3B), OI type III (Figure 3C), asphyxiating thoracic dystrophy (ATD), Ellis-van Creveld syndrome (EvC; Figure 4), caudal regression syndrome (CRS; Figure 5), congenital scoliosis (CS; Figure 6), spondylocostal dysostosis (SCD; Figure 7), and Klippel–Feil syndrome (KFS; Figures 8A and B). Demographic, clinical, and radiographic features of the cases are provided in Table 1. No case had regular antenatal visits; thus, prenatal diagnosis of abnormality was possible only toward the end of the pregnancies. In case 9, the mother was a diabetic and had a poor obstetric history. The majority of the cases had parental consanguinity (except cases 11 and 12). Nine cases were term, whereas 2 cases (cases 4 and 7) were near term. Case 12 was delivered at 31 weeks of gestational age. Except for cases 6, 7, 8, and 11, the remaining 8 cases required intubation in the delivery room.

**TABLE 1:**
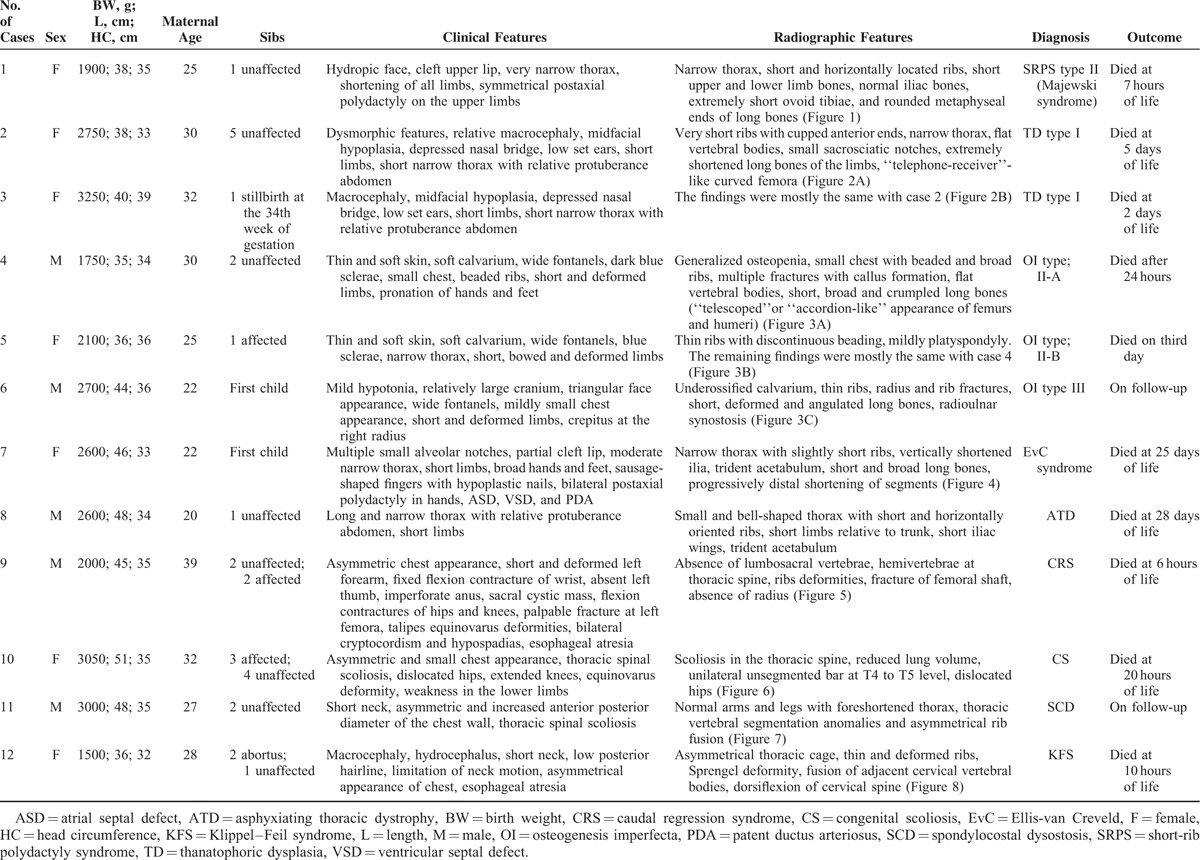
Demographic, Clinical, and Radiographic Features of Cases

Eight cases died within the first week of life due to cardiopulmonary insufficiency. Cases diagnosed with ATD and EvC syndrome were maintained on ventilatory support until their deaths at 4 weeks of life. The patient diagnosed with SCD was discharged with oxygen supplementation. He later required frequent rehospitalizations due to pneumonia and respiratory distress. He is currently waiting for surgery. After primary care and family education, the patient diagnosed with OI type III was discharged with a plan for regular clinical follow-ups of endocrinology, orthopedics, and physical therapy.

## DISCUSSION

Whereas advances in genetic research are clearly important in the diagnosis of skeletal dysplasias and in developing better therapy for those conditions, the role of the pediatrician as the diagnostician has remained unchanged.^8–11^ Because the widespread use of molecular genetic testing for bone dysplasias is not available in many areas of developing countries, the clinico-radiologic diagnosis is emphasized; distinction among the various bone dysplasias is based largely on clinical and radiographic findings.^8,9^ The radiologic evaluation begins with a complete skeletal survey, ideally composed of a set of radiographs,^8^ but because this study was designed retrospectively, the diagnoses of cases were achieved by limited radiography.

As seen in this small series, some bone dysplasias are lethal.^4^ A correct diagnosis and typing of the skeletal disorder is essential for the prognosis as is genetic counseling of the family, including the possibility of prenatal diagnosis in subsequent pregnancies.^10^ A number of these disorders (SRPS, EvC syndrome, ATD) are autosomal recessive inheritance, whereas some (OI type II and III, TD type I, achondroplasia), and most de novo mutations, are autosomal dominant.^1,9^ The recurrence risk for future pregnancies is close to 25% in autosomal recessive disorders.^10^ However, it is never 0 for the others, because of de novo mutation. An empiric recurrence risk is estimated as 2% to 3% among siblings resulting from parental mosaicism for the mutation that is lethal in the infant who is heterozygous for the same mutation or due to the rare recessive form.^10,12^ In this series, the high rate of consanguineous marriages and irregularity of antenatal visits, despite the presence of family history, indicate the lack of competent parental counseling. Additionally, live-born infants affected with perinatally lethal skeletal dysplasias are usually the offspring of families with a low socioeconomic background who have not received prenatal follow-up.

Because sonography has become a routine component of prenatal care, many of these disorders are diagnosed prenatally.^6,7^ The ability to achieve the correct specific diagnosis by prenatal ultrasound depends on the type of skeletal dysplasia and gestational age at which ultrasound is performed.^6,7^ Discriminating between lethal and nonlethal forms of skeletal dysplasias is of major clinical importance. Sonographic markers of lethality are mainly based on the assessment of lung biometry, measurements of chest circumference and its relation to the abdominal circumference, femur length, and, finally, assessment of the pulmonary arteries by Doppler ultrasonography.^6,7^ However, the accuracy of the specific diagnosis is still dependent on the molecular genetic or post mortem examination.^5^

### Differential Diagnosis

Short-rib dysplasias (with or without polydactyly) include SRPS (SRPS I–IV), ATD, and EvC syndrome.^1^ SRPS types 1 through 4 are lethal in the newborn period because of the severe pulmonary hypoplasia and other associated anomalies.^13–15^ On the other hand, the EvC syndrome and ATD are not uniformly lethal.^16,17^ Accurate prenatal diagnosis is important to provide adequate counseling.^13^

Overlap in the clinical and radiological features of SRPS types has led to difficulties in distinguishing between them. Type 1 SRPS (OMIM 613091) differs in shape (long bones with a torpedo-shape appearance) from type 3 SRPS (banana-peel shape), although there is a certain overlap of these features in the 2 disorders. In type 2 (OMIM 263520) and type 4 (OMIM 269860), the pelvis has a more normal appearance and the ends of the tubular bones are smooth. The occurrence of a median cleft lip also identifies type 2 and type 4 SRPS. However, short ovoid tibia are not seen in type 4 SRPS; this is the diagnostic finding of type 2 SRPS.^13–15^

Ellis-van Creveld syndrome (OMIM 225500) is characterized by progressively acromelic and mesomelic limb shortening, with smooth rounded metaphyses, postaxial polydactyly, small chest, ectodermal dysplasia, and, in many cases, congenital heart defects.^16^ The ribs are usually shorter in ATD (OMIM 208500), and heart defects and ectodermal dysplasia are not characteristics of ATD.^17^ The association of a small thorax with short ribs and a small pelvis is also seen in Barnes syndrome (OMIM 187760), which is distinguished from ATD by the presence of laryngeal stenosis and the absence of long bone changes and iliac spurs in infancy.^18^

Short-rib polydactyly syndrome should also be differentiated from other bone dysplasias presenting with micromelia and narrow thorax, namely achondrogenesis, TD, hypophosphatasia, and OI type II. These disorders are differentiated by their spinal, pelvic, and long bone changes.^9,10^ However, postaxial polydactyly is present only in SRPS.^13^

In achondrogenesis (OMIM 200600), there are unossified vertebral bodies, short ribs with splayed ends, hypoplastic ilia, and short, misshapen tubular bones with minimal tubulation.^10,19^ Infantile hypophosphatasia (OMIM 241500) is associated with severe demineralization and metaphyseal ossification defects reaching far into the diaphyses (the tubular bones are short and bowed with V-shaped ossification defects at their ends reaching deep into the diaphyses).^10,20^ Metaphyseal cupping and fraying of tubular bones have also been observed in Jansen metaphyseal dysplasia (OMIM 156400), which differs by the presence of splayed rib ends and normal alkaline phosphatase.^9,10^ In contrast to infantile hypophosphatasia, life expectancy is normal in Jansen metaphyseal dysplasia.^10^ OI type II (OMIM 166210) differs by the presence of rib and long bone fractures, and the absence of metaphyseal lesions.^21^ Flat vertebral bodies, squared iliac wings with wide, horizontal inferior margins, and curved femora with radiolucent upper ends in TD (OMIM 187600) rule out other lethal chondrodysplasia.^10,22^ A radiologically similar disease with favorable outcome is achondroplasia (OMIM 100800). The appearance of the vertebral bodies, pelvis, and tubular bones in achondroplasia is similar but milder than those in TD.^10^

The other rare skeletal dysplasias associated with narrow thorax and micromelia include campomelic dysplasia (OMIM 114290; bowed femora and tibia, normal bone density, hypoplastic scapula and vertebrae, and eleven pairs of ribs), Schneckenbecken dysplasia (OMIM 269250; short ribs with splayed ends, small ilia with medial projection from the inner margins, shortened, dumbbell-shaped tubular bones), fibrochondrogenesis (OMIM 228520; radiologically similar to Schneckenbecken dysplasia, small ilia with spurs extending caudally from the acetabular roof), metatropic dysplasia (OMIM 156530; mushrooming of the long tubular bones), and lethal metaphyseal chondrodysplasia (OMIM 250220; short ribs with splayed posterior ends and cupped anterior ribs ends, mildly shortened tubular bones with metaphyseal cupping and irregularity).^9,10^

Osteogenesis imperfecta type II is characterized by early prenatal onset of severe bone shortening and bowing of the long bones due to multiple fractures, poor demineralization of the skull, and a narrow and bell-shaped chest caused by fractures of the ribs.^21,23^ OI type II has been divided into OI types II-A, II-B, and II-C on the base of radiological characteristics.^21^ OI type II differs in the shortened, crumpled long bones, especially compressed (“telescoped” or “accordion-like”) femurs and/or humeri, and continuous beaded and broad ribs from OI type III (OMIM 259420), which has thin ribs with rare fractures and markedly angulated long bones, with wide metaphyses and thinner diaphyses.^10,21^ Although OI type III is not uniformly lethal in early infancy, as seen in this study, due to continuing fractures after birth, the appearance of the long bones can change in the course of time to the thick tubular bones seen in newborns with type II bone changes.^10,21^ In OI type III, thoracic insufficiency is also evident in later periods.^21^

Congenital scoliosis is a skeletal disorder in which lateral curvature of the skeletal spine results from asymmetric biomechanical forces, and can occur with many conditions including abnormal vertebral segmentation, neuromuscular disorders, and rare congenital syndromes.^24^ Therefore, it may be described as a clinical finding rather than a precise diagnosis. Severe CS may produce a progressive loss of torso mobility, resulting in fixed postural asymmetry, reduction in chest wall movement, and a consequent pulmonary hypoplasia and thoracic insufficiency.^24^

Caudal regression syndrome (OMIM 600145) is a rare and often sporadic congenital malformation of the lower vertebral column, characterized by partial or complete absence of sacrum and lumbar vertebrae.^25^ Associations most often reported with CRS include genitourinary, anorectal, vertebral, and cardiopulmonary anomalies, and may also include VACTERL association (OMIM 192350; abnormality of vertebrae, anus, cardiovascular system, trachea, esophagus, renal system, and limb buds), and sirenomelia (fusion of the lower limbs).^25,26^ Respiratory problems may arise from abnormal chest shape and size.^25^

Spondylocostal dysostosis is one of the 2 forms of Jarcho–Levin syndrome (OMIM 277300), which is characterized by distinctive malformations of bones of the spinal column (vertebrae) and the ribs, respiratory insufficiency, and/or other abnormalities.^27^ It is important to distinguish between SCD and spondylothoracic dysplasia (STD), because survival may be possible in the former, but the latter is usually fatal.^27^ SCD is differentiated by vertebral malformations, frequent dramatic rib malformations, and the absence of fan-like or crab-like thoracic configuration, whereas STD is characterized by vertebral body malformations and ribs that flare in a fan-like pattern, but that are not significantly malformed.^27^ The differential diagnosis of a patient with vertebral and rib anomalies includes KFS (OMIM 118100; cervical vertebral fusion, Sprengel deformity of the shoulder),^28^ dyssegmental dysplasia (OMIM 224400; micromelia with reduced mobility, bowing of the long bone with dumbbell-shaped metaphyses), CS, VATER/VACTERL, and COVESDEM (OMIM 268310; costovertebral segmentation defects with mesomelia) associations.

## CONCLUSIONS

The clinical findings and examination of the skeletal radiographs permit precise diagnoses in the majority of cases with skeletal dysplasias, since the classification of skeletal dysplasias is largely based on clinico-radiographic findings. Knowledge of the main findings allows us to make the differential diagnosis. The high rate of consanguineous marriages and the irregularity of antenatal visits, plus the presence of a family history of skeletal dysplasias, emphasize the importance of both detailed and persistent parental counseling.

## References

[1] Bonafe L Cormier-Daire V Hall C Nosology and classification of genetic skeletal disorders: 2015 revision. *Am J Med Genet* 2015; 167:2869–2892. 26394607 10.1002/ajmg.a.37365

[2] Orioli IM Castilla EE Barbosa-Neto JG The birth prevalence rates for skeletal dysplasias. *J Med Genet* 1986; 23:328–332. 3746832 10.1136/jmg.23.4.328 PMC1049699

[3] Barbosa-Buck CO Orioli IM da Graça Dutra M Clinical epidemiology of skeletal dysplasias in South America. *Am J Med Genet A* 2012; 158A:1038–1045. 22407836 10.1002/ajmg.a.35246

[4] Camera G Mastroiacovo P Birth prevalence of skeletal dysplasias in the Italian multicentric monitoring system for birth defects. *Prog Clin Biol Res* 1982; 104:441–449. 7163285

[5] Anastasia Konstantinidou. Skeletal dysplasias of the human fetus: postmortem diagnosis. In: Choy R (ed.). Prenatal Diagnosis: Morphology Scan and Invasive Methods. 2012; ISBN: 978–953–51–0614–2.

[6] Parilla BV Leeth EA Kambich MP Antenatal detection of skeletal dysplasias. *J Ultrasound Med* 2003; 22:255–258. 12636325 10.7863/jum.2003.22.3.255

[7] Schramm T Gloning KP Minderer S Prenatal sonographic diagnosis of skeletal dysplasias. *Ultrasound Obstet Gynecol* 2009; 34:160–170. 19548204 10.1002/uog.6359

[8] Panda A Gamanagatti S Jana M Skeletal dysplasias: a radiographic approach and review of common non-lethal skeletal dysplasias. *World J Radiol* 2014; 6:808–825. 25349664 10.4329/wjr.v6.i10.808 PMC4209426

[9] Alanay Y Lachman RS A review of the principles of radiological assessment of skeletal dysplasias. *J Clin Res Pediatr Endocrinol* 2011; 3:163–178. 22155458 10.4274/jcrpe.463 PMC3245489

[10] Schumacher R Seaver LH Spranger JW Fetal Radiology: A Diagnostic Atlas. 2nd ed. Berlin Heidelberg: Springer; 2010.

[11] Mortier GR The diagnosis of skeletal dysplasias: a multidisciplinary approach. *Eur J Radiol* 2001; 40:161–167. 11731205 10.1016/s0720-048x(01)00397-7

[12] Pyott SM Pepin MG Schwarze U Recurrence of perinatal lethal osteogenesis imperfecta in sibships: parsing the risk between parental mosaicism for dominant mutations and autosomal recessive inheritance. *Genet Med* 2011; 13:125–130. 21239989 10.1097/GIM.0b013e318202e0f6

[13] Jutur PS Kumar CP Goroshi S Case report: short rib polydactyly syndrome: type 2 (Majewski syndrome). *Indian J Radiol Imaging* 2010; 20:138–142. 20607029 10.4103/0971-3026.63044 PMC2890924

[14] Sarafoglou K Funai EF Fefferman N Short rib-polydactyly syndrome: more evidence of a continuous spectrum. *Clin Genet* 1999; 56:145–148. 10517252 10.1034/j.1399-0004.1999.560209.x

[15] Naki MM Gür D Zemheri E Short rib-polydactyly syndrome. *Arch Gynecol Obstet* 2005; 272:173–175. 15605271 10.1007/s00404-004-0696-9

[16] Baujat G Le Merrer M Ellis-van Creveld syndrome. *Orphanet J Rare Dis* 2007; 2:27. 17547743 10.1186/1750-1172-2-27 PMC1891277

[17] Oberklaid F Danks DM Mayne V Asphyxiating thoracic dysplasia. Clinical, radiological, and pathological information on 10 patients. *Arch Dis Child* 1977; 52:758–765. 931421 10.1136/adc.52.10.758 PMC1544803

[18] Burn J Hall C Marsden D Autosomal dominant thoracolaryngopelvic dysplasia: Barnes syndrome. *J Med Genet* 1986; 23:345–349. 3746836 10.1136/jmg.23.4.345 PMC1049703

[19] Borochowitz Z Lachman R Adomian GE Achondrogenesis type I: delineation of further heterogeneity and identification of two distinct subgroups. *J Pediatr* 1988; 112:23–31. 3275766 10.1016/s0022-3476(88)80113-6

[20] Mornet E Hypophosphatasia. *Best Pract Res Clin Rheumatol* 2008; 22:113–127. 18328985 10.1016/j.berh.2007.11.003

[21] van Dijk FS Cobben JM Kariminejad A Osteogenesis imperfecta: a review with clinical examples. *Mol Syndromol* 2011; 2:1–20. 22570641 10.1159/000332228 PMC3343766

[22] Kölble N Sobetzko D Ersch J Diagnosis of skeletal dysplasia by multidisciplinary assessment: a report of two cases of thanatophoric dysplasia. *Ultrasound Obstet Gynecol* 2002; 19:92–98. 11851976 10.1046/j.0960-7692.2001.00496.x

[23] Renaud A Aucourt J Weill J Radiographic features of osteogenesis imperfecta. *Insights Imaging* 2013; 4:417–429. 23686748 10.1007/s13244-013-0258-4 PMC3731461

[24] Brand MC Examination of the newborn with congenital scoliosis: focus on the physical. *Adv Neonatal Care* 2008; 8:265–273. 10.1097/01.ANC.0000338016.03040.6b 18827515

[25] Boulas MM Recognition of caudal regression syndrome. *Adv Neonatal Care* 2009; 9:61–69. 19363325 10.1097/ANC.0b013e31819de44f

[26] Singh SK Singh RD Sharma A Caudal regression syndrome: case report and review of literature. *Pediatr Surg Int* 2005; 21:578–581. 15977017 10.1007/s00383-005-1451-4

[27] Berdon WE Lampl BS Cornier AS Clinical and radiological distinction between spondylothoracic dysostosis (Lavy-Moseley syndrome) and spondylocostal dysostosis (Jarcho-Levin syndrome). *Pediatr Radiol* 2011; 41:384–388. 21174082 10.1007/s00247-010-1928-8

[28] Tracy MR Dormans JP Kusumi K Klippel-Feil syndrome: clinical features and current understanding of etiology. *Clin Orthop Relat Res* 2004; 424:183–190. 15241163

